# A Novel Inhibitor of STAT5 Signaling Overcomes Chemotherapy Resistance in Myeloid Leukemia Cells

**DOI:** 10.3390/cancers11122043

**Published:** 2019-12-17

**Authors:** Marie Brachet-Botineau, Margaux Deynoux, Nicolas Vallet, Marion Polomski, Ludovic Juen, Olivier Hérault, Frédéric Mazurier, Marie-Claude Viaud-Massuard, Gildas Prié, Fabrice Gouilleux

**Affiliations:** 1 LNOx, GICC, CNRS ERL 7001, University of Tours, 37000 Tours, France; marie.brachet@univ-tours.fr (M.B.-B.); margaux.deynoux@etu.univ-tours.fr (M.D.); nls.vallet@gmail.com (N.V.); olivier.herault@univ-tours.fr (O.H.); frederic.mazurier@inserm.fr (F.M.); 2 Service d’Hématologie et Thérapie Cellulaire, CHRU de Tours, 37000 Tours, France; 3 IMT, GICC, EA 7501, University of Tours, 37000 Tours, France; marion.polomski@etu.univ-tours.fr (M.P.); ludovic.juen@mcsaf.fr (L.J.); marie-claude.viaud-massuard@univ-tours.fr (M.-C.V.-M.); gildas.prie@univ-tours.fr (G.P.); 4 Service d’Hematologie Biologique, CHRU de Tours, 37000 Tours, France

**Keywords:** pharmacological inhibitor, STAT5 signaling, chemotherapy resistance, myeloid leukemia

## Abstract

Signal transducers and activators of transcription 5A and 5B (STAT5A and STAT5B) are crucial downstream effectors of tyrosine kinase oncogenes (TKO) such as BCR-ABL in chronic myeloid leukemia (CML) and FLT3-ITD in acute myeloid leukemia (AML). Both proteins have been shown to promote the resistance of CML cells to tyrosine kinase inhibitors (TKI) such as imatinib mesylate (IM). We recently synthesized and discovered a new inhibitor (17f) with promising antileukemic activity. 17f selectively inhibits STAT5 signaling in CML and AML cells by interfering with the phosphorylation and transcriptional activity of these proteins. In this study, the effects of 17f were evaluated on CML and AML cell lines that respectively acquired resistance to IM and cytarabine (Ara-C), a conventional therapeutic agent used in AML treatment. We showed that 17f strongly inhibits the growth and survival of resistant CML and AML cells when associated with IM or Ara-C. We also obtained evidence that 17f inhibits STAT5B but not STAT5A protein expression in resistant CML and AML cells. Furthermore, we demonstrated that 17f also targets oncogenic STAT5B N642H mutant in transformed hematopoietic cells.

## 1. Introduction

STAT5A and STAT5B are two closely related signal transducers and activators of transcription family members. Both proteins are crucial downstream effectors of tyrosine kinase oncogenes (TKO) such as Fms-like receptor tyrosine kinase 3 with internal tandem duplications (Flt3-ITD), BCR-ABL and JAK2^V617F^ which cause AML, CML and other myeloproliferative diseases (MPD), respectively [1]. STAT5 proteins are recognized as major drivers in the development and/or maintenance of CML as well as in the proliferation and survival of AML cells [2,3,4]. The development of tyrosine kinase inhibitors (TKI) targeting BCR-ABL such as imatinib mesylate (IM) has revolutionized the treatment of CML. Despite this success story, IM is not totally curative and approximately 50% of patients remain therapy-free after IM discontinuation. The inability of IM to completely eradicate leukemic stem cells (LSC) is probably responsible for the relapse of CML patients [5]. Moreover, the occurrence of *BCR-ABL* mutations in progressive or relapsed disease promotes IM resistance of CML cells [6]. Therefore, there is a need for complementary therapeutic strategies to cure CML. STAT5 fulfils all the criteria of a major drug target in CML [7]. High STAT5 expression levels have been shown not only to enhance IM resistance in CML cells but also to trigger *BCR*-*ABL* mutations by inducing the production of reactive oxygen species (ROS) responsible for DNA damage [8,9]. Moreover, STAT5 was shown to play a key role in the maintenance of chemoresistant CML stem cells [10]. Thus, targeting STAT5 would also benefit relapsed CML patients who became resistant to TKI. Several approaches have been used to target STAT5 in leukemia. Among them, cell-based screening with small molecule libraries of already approved drugs allowed the identification of the psychotropic drug pimozide as a potential STAT5 inhibitor in CML cells [11]. Pimozide decreased the tyrosine phosphorylation of STAT5 and induced growth arrest and apoptosis in CML cells. In addition, pimozide was shown to target the deubiquitinating (DUB) enzyme, USP1, in leukemic cells indicating that the effects of pimozide on STAT5 activity might be indirect [12]. Indirubin derivatives were also reported to inhibit STAT5 phosphorylation in CML cells but the mechanism of inhibition is most likely suppression of upstream tyrosine kinases [13]. More recently, a number of small inhibitors that bind to the Src homology domain 2 (SH_2_) required for STAT5 activation and dimer formation, have been described [14]. These compounds exhibit potent and selective binding activity for STAT5 by effectively disrupting phosphopeptide interactions. Some of these inhibitors bind STAT5 proteins in a nanomolar range and inhibit the tyrosine phosphorylation of STAT5 and CML/AML cell growth in a micromolar range [15,16,17]. A final approach is to target STAT5 activity through the activation of peroxisome proliferator-activated receptor gamma (PPARγ) [18]. Indeed, the existence of cross-talk between PPARγ and STAT5 has been discussed. For instance, antidiabetic drugs such as glitazones, which are PPARγ agonists, were shown to have antileukemic activity [19,20]. Activation of PPARγ by pioglitazone not only decreases the phosphorylation of STAT5 in CML cells but also reduces expression of *STAT5* genes in quiescent and resistant CML stem cells [10]. Importantly, the combined use of pioglitazone and IM triggers apoptosis of these leukemic cells suggesting that besides phosphorylation, inhibition of STAT5 expression is of prime importance for resistant CML stem cell eradication. Based on these different data, we sought to identify new STAT5 inhibitors in a library of PPARα/γ ligands that were synthetized in our laboratory [21,22]. The synthesis of derivatives of a “hit” compound identified in the library screening allowed the discovery of a new inhibitor of STAT5 signaling in CML and AML cells [23]. This molecule (17f) selectively inhibits the phosphorylation and transcriptional activity of STAT5 and induces apoptosis of CML and AML cells. Herein, we showed that 17f associated with IM or Ara-C resensitizes CML and AML cells, respectively, that acquired resistance to these drugs. We demonstrated that 17f treatment reduces STAT5B protein levels in resistant CML and AML cells, suggesting that 17f overcomes chemotherapy resistance though the downregulation of this protein. We also found that 17f suppresses expression of oncogenic STAT5^N642H^ mutant in transformed Ba/F3 cells. 

## 2. Results

### 2.1. Effects of 17f Compound on Growth and Viability of IM-Sensitive and IM-Resistant BCR-ABL^+^ Cells

**Figure 1:**
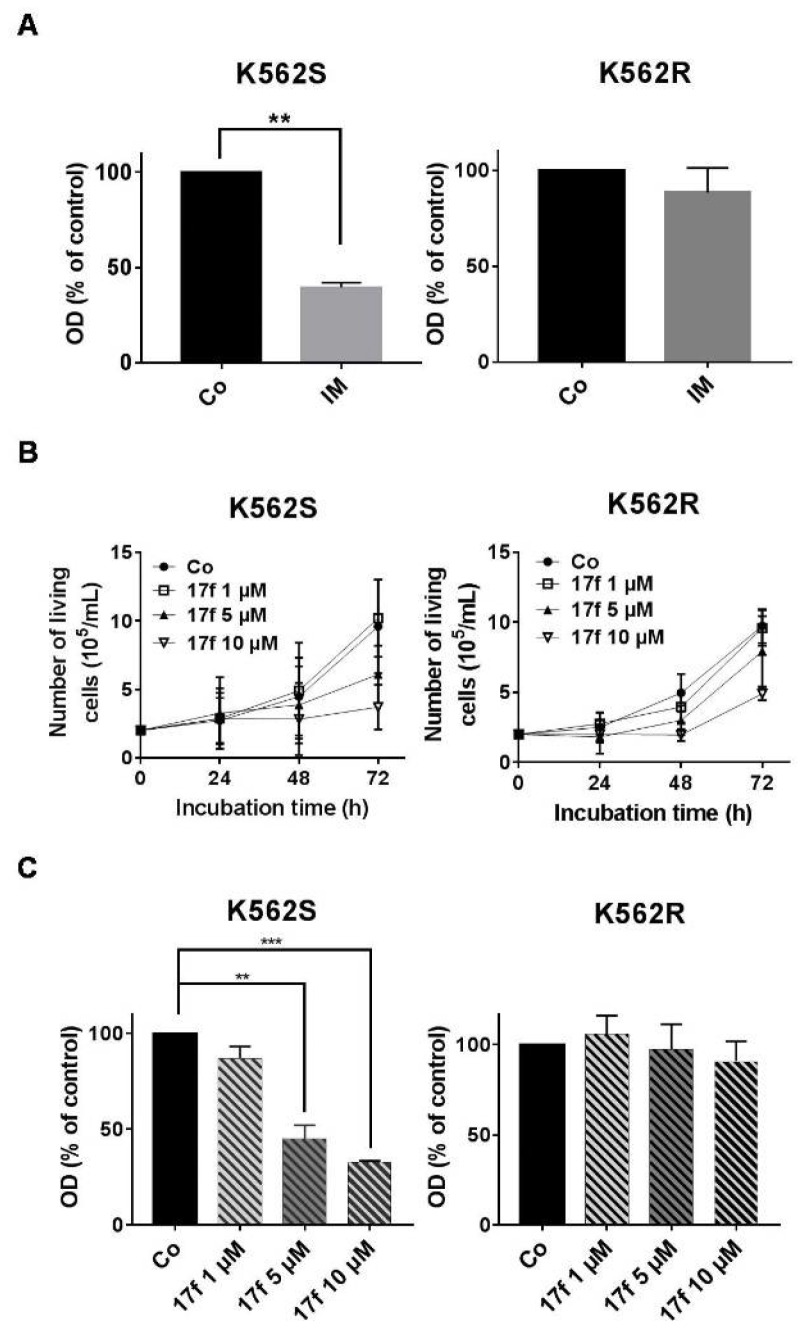
Effects of 17f molecule on K562S and K562R cell growth (**A**) Imatinib mesylate (IM)-sensitive K562 (K562S) and IM-resistant K562 cells (K562R) were treated with 1 µM IM or DMSO as control (Co) for 48 h. Cell viability was determined by MTT assays (data are presented as mean ± SD of three independent experiments (*n* = 3) in triplicates, *** *p* < 0.001; one sample *t*-test). (**B**) K562S and K562R cells were treated with 17f or DMSO as control (Co) for the indicated times. Growth kinetics were determined by trypan blue dye exclusion assays (*n* = 3 in triplicates, data are mean ± SD). (**C**) Cell viability was measured by MTT assays after treatment of K562S or K562R cells with increasing concentrations of 17f or DMSO as control (Co) during 48 h (*n* = 3 in triplicates, data are mean ± SD, ** *p* < 0.01, *** *p* < 0.001; one sample *t*-test).

Initial experiments were carried out to determine the effects of 17f alone (see structure in Figure S1) on K562 cells that are sensitive (K562S) or resistant (K562R) to IM treatment. These in vitro models are depicted in Figure 1A. Sensitive and resistant cells were treated with various concentrations of 17f (ranging from 1 to 10 µM). Growth and viability were determined by trypan blue exclusion (Figure 1B) and MTT (3-(4,5-dimethylthiazol-2-yl)-2,5-diphenyltetrazolium bromide) (Figure 1C) assays. Addition of 17f clearly blocked the growth of K562S cells while K562R cells remain insensitive to 17f treatment at the same concentration. The EC_50_ value was found to be two times higher in K562R cells than in K562S cells (14.5 ± 4.8 µM vs. 6.9 ± 1.7 µM). We also observed that treatment with 5 µM 17f did not affect the growth and viability of K562R cells and used this suboptimal concentration in most experiments to evaluate the combined effects of 17f and IM.

### 2.2. 17f Induces Apoptosis and Cell Cycle Arrest in K562R Cells and Relieves the Resistance to IM

**Figure 2:**
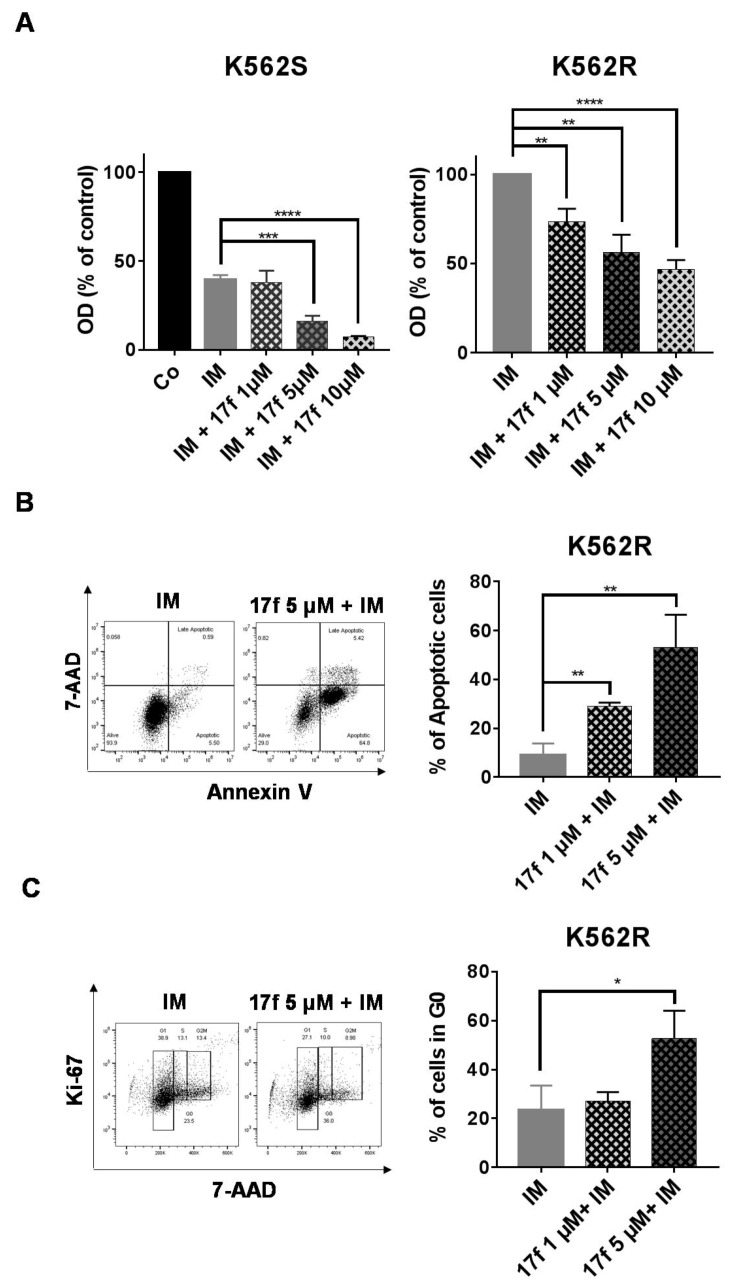
17f overcomes the resistance of K562R cells to IM treatment. (**A**) K562S and K562R cells were treated with IM or not (Co) with or without 17f for 48 h. Cell viability was determined by MTT assays. (**B**) K562R cells cultured for 48 h with IM and 17f or IM vs. DMSO as control. Cells were stained with anti-annexin V coupled with FITC (fluorescein isothiocyanate) and with 7-amino-actinomycin D (7-AAD) to determine the percentages of apoptotic cells. One representative experiment is shown (left panel). (**C**) K562R cells treated for 48 h with IM and 17f or IM and DMSO as control were stained with 7-AAD and an Alexa Fluor 488-conjugated anti-Ki-67 antibody. Cell cycle phase distributions were then estimated by flow cytometry. The histogram presents the percentage of cells in the G_0_ phase. One representative experiment is shown (left panel) (*n* = 3 in triplicates, data are mean ± SD, * *p* < 0.05, ** *p* < 0.01, *** *p* < 0.001, **** *p* < 0.0001).

We then addressed whether 17f in combination with IM might directly abrogate the resistance of K562R cells to IM. K562S and K562R cells were treated with 17f in the presence of IM and cell growth and viability were determined by MTT assays (Figure 2A). As expected, IM strongly inhibited the growth of K562S cells. This inhibition was further enhanced by 17f in a dose-dependent fashion. Interestingly, we found that the addition of 1 µM 17f in the presence of IM was already enough to significantly reduce the growth and viability of K562R cells. Treatment with 5 µM and 10 µM of 17f further increased this inhibitory effect. To analyze the growth-suppressive properties of 17f in K562R cells, we determined the impact of this small molecule on apoptosis and the cell cycle. 17f induced apoptosis and changes in cell cycle phase distribution in a concentration-dependent manner (Figure 2B,C). 17f significantly increased the number of cells in the G_0_ phase indicating that treatment with this compound induced quiescence of K562R cells.

### 2.3. 17f Inhibits STAT5-Dependent Transcriptional Activity in K562R Cells

**Figure 3:**
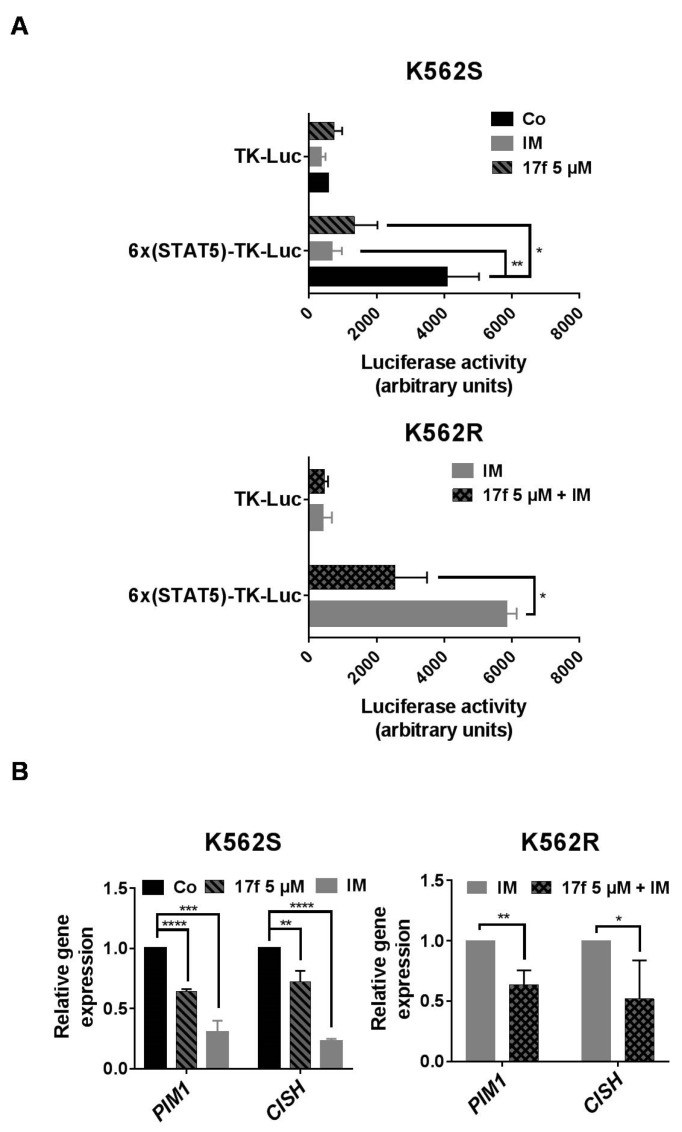
17f associated with IM inhibit STAT5 activity in resistant K562R cells. (**A**) K562S or K562R cells transfected with a 6×(STAT5)-TK-luciferase reporter construct or a control TK-luciferase vector were treated or not (Co) with 17f (5 µM), IM (1 µM) or with the combination of 17f and IM for 48 h. Luciferase activities were then determined as described in Methods. Luciferase activity (arbitrary units) in the histogram represents the relative luminescence unit (rlu) values/mg of proteins (*n* = 3 in triplicates, data are mean ± SD, * *p* < 0.05, ** *p* < 0.001). (**B**) qRT-PCR analysis of *PIM1* and *CISH* expression in K562S and K562R treated or not (Co) with IM (1 µM),17f (5 µM) or with combined 17f and IM for 24 h. Results are presented as the fold change in *PIM1* and *CISH* gene expression in treated cells normalized to internal control genes (*GAPDH*, *ACTB* and *RPL13a*) and relative to control condition (normalized to 1) (*n* = 3 in triplicates, data are mean ± SD, * *p* < 0.05; one-sample *t*-test).

We previously showed that 17f inhibits the transcriptional activity of STAT5 in CML cells. We then asked whether this small molecule also affects the activity of these proteins in IM-resistant K562R cells. We first determined the impact of this compound on the transcriptional activation of a reporter gene driven by a STAT5-specific promoter. K562S and K562R cells were transfected with a construct containing six tandem copies of the STAT5 response element in front of the minimal TK promoter fused to the luciferase reporter gene (6×(STAT5)-TK-luc). As control, cells were also transfected with a TK-luciferase vector without STAT5 response elements (TK-luc). Luciferase activity was determined 48 h post-transfection in K562S and K562R cells treated with DMSO as control, 17f (5 µM) and/or IM (1 µM). As expected, constitutive STAT5 activity induced by BCR-ABL increased luciferase activity in K562S cells transfected with the STAT5-dependent promoter construct compared to cells transfected with the control TK-luc vector (Figure 3A). This enhanced luciferase activity was strongly reduced after 17f or IM treatment. In sharp contrast, the luciferase activity remained elevated after treatment with IM in K562R cells transfected with the STAT5-dependent reporter construct, although this enzymatic activity was strongly decreased after the addition of 17f and IM. qRT-PCR experiments were then conducted to determine the effects of 17f on STAT5-dependent expression of target genes such as *PIM1* and *CISH* (Figure 3B). As expected, 17f or IM reduced expression of both genes in sensitive K562S cells while this effect was observed in resistant K562R cells after treatment with both compounds. Collectively, these data strongly suggest that 17f inhibits the transcriptional activity of STAT5 to bypass IM resistance in K562R cells.

### 2.4. 17f Inhibits STAT5B Protein Expression in IM-Resistant K562 Cells

**Figure 4:**
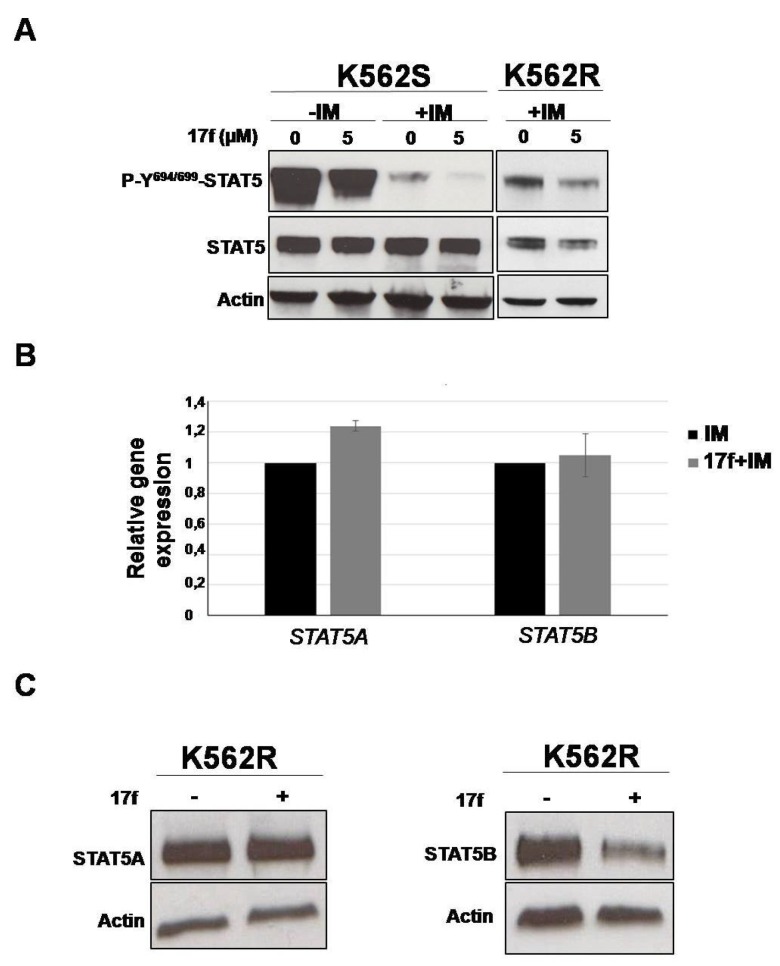
17f associated with IM inhibits STAT5B protein expression in K562R cells (**A**) Protein extracts from K562S and K562R cells treated with 17f 5 µM or DMSO with or without IM for 24 h were analyzed by western blotting to detect P-Y^694/699^-STAT5 and STAT5 protein expression (*n* = 2). Actin served as the loading control. (**B**) qRT-PCR analysis of *STAT5A* and *STAT5B* expression in K562R cultured with IM (1 µM) as control or treated with 17f (5µM) and IM for 24 h. Results are presented as the fold change in *STAT5A* and *STAT5B* gene expression in treated cells normalized to internal control genes (*GAPDH*, *ACTB* and *RPL13a*) and relative to control condition (normalized to 1) (*n* = 3 in triplicates, data are mean ± SD, * *p* < 0.05; one sample *t*-test). (**C**) Expression of STAT5A and STAT5B proteins in K562R cells treated or not with 17f (5 µM) was analyzed by western blot (*n* = 2). Actin served as the loading control.

We then determined the impact of 17f on BCR-ABL-induced tyrosine phosphorylation of STAT5 (P-Y^694/699^-STAT5) by western blot and flow cytometry analysis (Figure 4A, Figures S2–S4). K562S and K562R cells were treated for 24 h instead of 48 h to analyze the early effects on STAT5 phosphorylation. IM strongly reduced P-Y-STAT5 levels in K562S, and the addition of 17f further enhanced this effect. P-Y-STAT5 levels were maintained in IM-treated K562R cells but were decreased after the addition of 17f. Interestingly, the level of STAT5 phosphorylation was strikingly enhanced in K562R cells after removal of IM and was weakly affected by the addition of 17f (Figure S5). To determine whether changes in P-Y-STAT5 levels reflect differences in protein abundance, immunoblots were performed with an anti-STAT5 antibody that recognizes STAT5A and STAT5B proteins (Figure 4B and Figure S2). As expected, IM inhibited the phosphorylation of STAT5 in sensitive and resistant cells. Interestingly, we observed that the association of 17f with IM reduces STAT5 expression in K562R cells but not in K562S cells (see Figure S3A,B for quantification). qRT-PCR experiments were then conducted to evaluate the impact of combination treatments on *STAT5A* and *STAT5B* gene expression in K562R cells. Results showed that *STAT5A/5B* mRNA levels were not affected by 17f when associated with IM (Figure 4B). In contrast, western blot analysis clearly evidenced that STAT5B protein expression was decreased after combination treatments suggesting that 17f sensitizes K562R cells to IM treatment by targeting STAT5B protein (Figure 4C). 

### 2.5. Effects of 17f on Growth and Viability of Ara-C-Sensitive and Ara-C-Resistant FLT3-ITD Expressing Leukemic Cells

**Figure 5:**
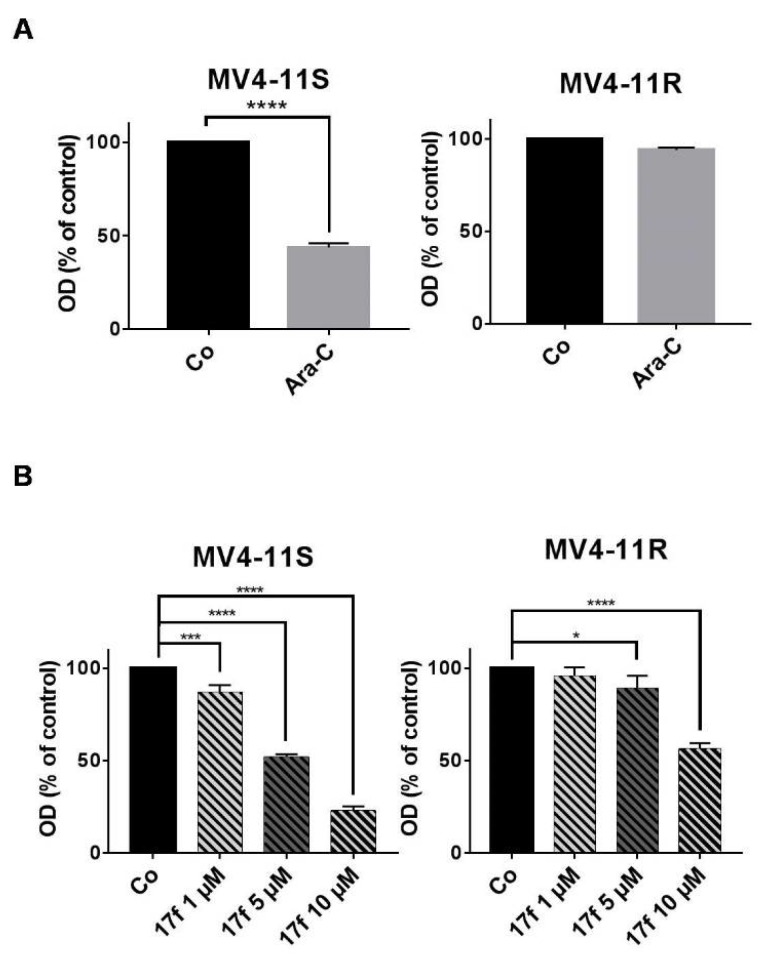
Effects of 17f on MV4-11S and MV4-11R cell growth (**A**) Ara-C-sensitive MV4-11 (MV4-11S) and Ara-C-resistant MV4-11 (MV4-11R) cells were treated with 1 µM Ara-C or DMSO as control (Co) for 48 h. Cell viability was then determined by MTT assays (*n* = 3 in triplicates, data are mean ± SD, **** *p* < 0.0001; one-sample *t*-test). (**B**) MV4-11S and MV4-11R cells were treated or not (Co) with increasing concentrations of 17f during 48 h. Cell viability was determined by MTT assays (*n* = 3 in triplicates, data are mean ± SD, ** *p* < 0.01, *** *p* < 0.001, **** *p* < 0.0001; one-sample *t*-test).

STAT5 is also phosphorylated by FLT3-ITD, a major TKO in AML cells. To exclude the possibility that 17f-mediated inhibition of STAT5 and cell growth is a peculiarity of IM-resistant BCR-ABL^+^ cells, we used MV4-11 cells expressing FLT3-ITD that acquired resistance to Ara-C, a conventional therapeutic agent that affects DNA replication. Sensitive and resistant MV4-11 cell models are depicted in Figure 5A. We first evaluated the impact of 17f alone on MV4-11S and MV4-11R cell growth and showed that MV4-11R cells were more resistant to 17f treatment than MV4-11S cells (Figure 5B). Based on these data, IC_50_ values were found to be three-fold higher in MV4-11R than in MV11-4S cells (10.79 ± 3.2 vs. 3.55 ± 0.47).

### 2.6. 17f Sensitizes MV4-11R Cells to Ara-C Treatment

**Figure 6:**
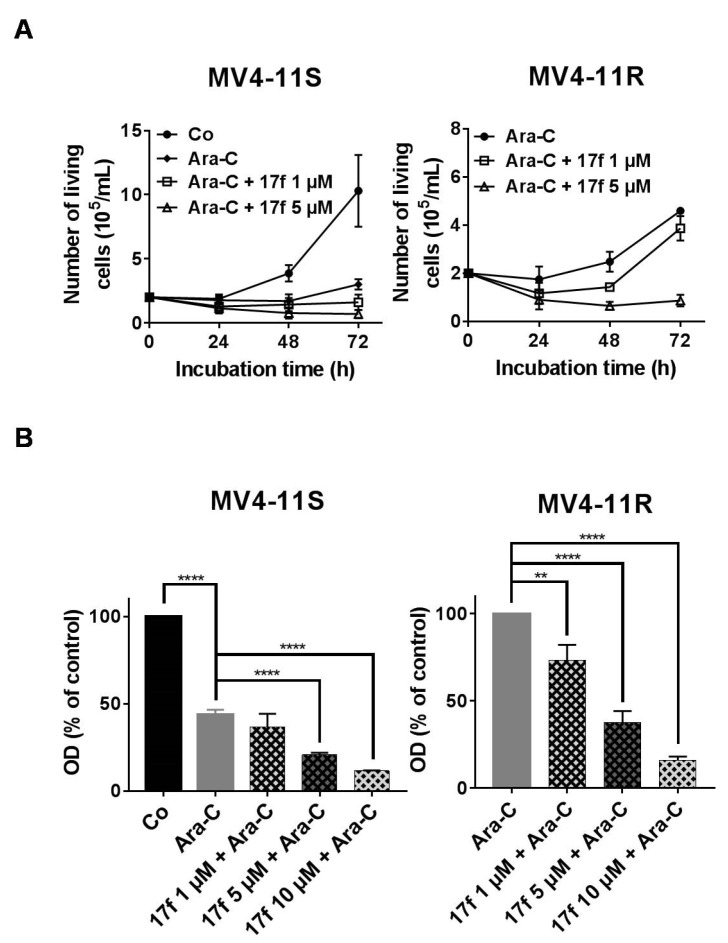
17f relieves the resistance of MV4-11R cells to ARA-C treatment. (**A**) MV4-11S or MV4-11R cells were treated with Ara-C or not (Co) with or without 17f. Growth kinetics were determined by Trypan blue dye exclusion assays (*n* = 3 in triplicates, data are mean ± SD). (**B**) MV4-11S or MV4-11R cells were treated with Ara-C or not (Co) with or without 17f for 48 h. Cell viability was determined by MTT assays (n = 3 in triplicates, data are mean ± SD, * *p* < 0.05, ** *p* < 0.01, **** *p* < 0.0001; one-sample *t*-test).

We then analyzed the effects of 17f on MV4-11S and MV4-11R cell growth in the presence of Ara-C using trypan blue dye exclusion (Figure 6A) and MTT assays (Figure 6B). Addition of 17f significantly enhanced the growth inhibition and cytotoxic effect of Ara-C in MV4-11S cells. Importantly, 17f greatly reduced the growth of resistant MV4-11R cells cultured with Ara-C in a concentration-dependent fashion. This growth inhibition was already observed with 1 µM, a concentration that did not affect the growth of MV4-11R cells cultured in the absence of Ara-C. These data indicated that the addition of 17f overcomes the resistance of MV4-11R cells to Ara-C. 

### 2.7. 17f Triggers Apoptosis, Cell Cycle Arrest and Inhibition of STAT5B Expression in MV4-11R Cells

**Figure 7:**
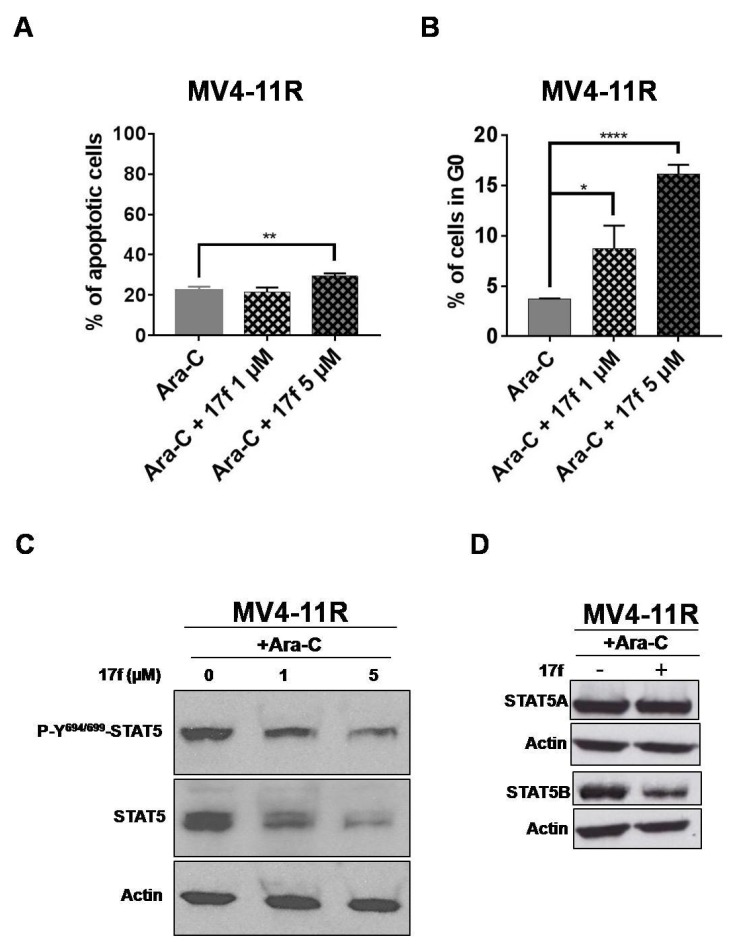
17f promotes apoptosis, cell cycle arrest and inhibition of STAT5B protein expression in MV4-11R cells. (**A**) Flow cytometry histogram of MV4-11R cells cultured for 48 h with Ara-C and 17f or Ara-C and DMSO as control. Cells were stained with anti-annexin V coupled with FITC and with 7-AAD to determine the percentages of apoptotic cells (*n* = 3 in triplicates, data are mean ± SD, ** *p* < 0.01). (**B**) MV4-11R cells treated for 48 h with Ara-C and 17f or Ara-C and DMSO as control were stained with 7-AAD and an Alexa Fluor 488-conjugated anti-Ki67 antibody. Cell cycle phase distributions were then estimated by flow cytometry. The histogram presents the percentage of cells in the G_0_ phase (*n* = 3 in triplicates, data are mean ± SD, * *p* < 0.05, *** *p* < 0.001). (**C**) Protein extracts from MV4-11R cells treated with Ara-C and 17f or Ara-C and DMSO for 24 h were analyzed by immunoblotting to detect P-Y^694/699^-STAT5 and STAT5 protein expression (*n* = 2). Actin served as the loading control. (**D**) Expression of STAT5A and STAT5B proteins in MV4-11R cells treated or not with 17f (5 µM) was also analyzed by western blot (*n* = 2).

We then evaluated the effects of 17f on apoptosis and the cell cycle in MV4-11R cells. A significant increase in apoptotic cells was observed (Figure 7A) only after treatment with 5 µM 17f, while the addition of 1 µM was enough to enhance the number of cells in the G_0_ phase of the cell cycle (Figure 7B). These results indicated that the growth-suppressive properties of 17f primarily affect the cell cycle in MV4-11R cells and apoptosis at higher concentrations. We then asked whether 17f interferes with STAT5 signaling in Ara-C-resistant AML cells and analyzed the impact of 17f on phosphorylation and expression of STAT5 in MV4-11R cells. In the absence of Ara-C, the level of STAT5 phosphorylation was slightly enhanced in MV4-11R cells (Figure S5C,D). The addition of 17f with or without Ara-C inhibited STAT5 expression in MV4-11R cells (Figure 7C and Figures S2B and S3C). Likewise, STAT5B expression was reduced after treatment with 17f alone or with Ara-C in resistant cells (Figure 7D). 

### 2.8. 17f Inhibits Expression of Oncogenic STAT5B^N642H^ Mutant

**Figure 8:**
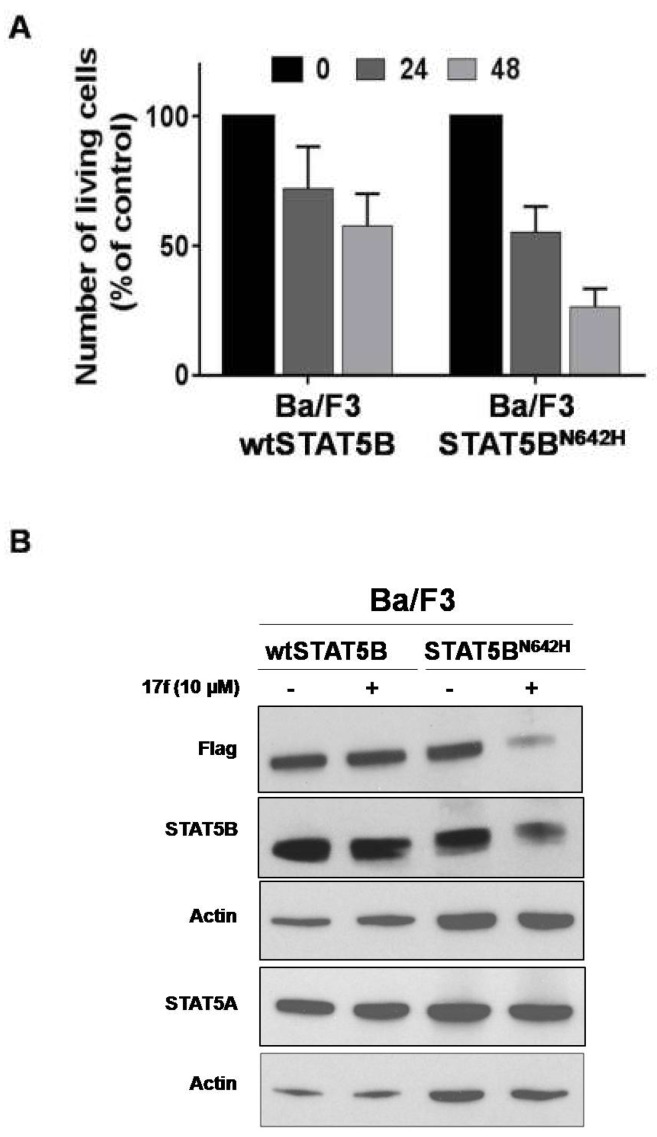
17f inhibits STAT5B^N642H^ activity and expression in Ba/F3 cells. (**A**) Cells were treated or not with 17f (10 µM). Growth were then determined by Trypan blue dye exclusion assays at the indicated times (*n* = 5 in triplicates, data are mean ± SD). (**B**) Protein extracts from MV4-11R cells treated with 17f for 24 h were analyzed by immunoblotting to detect flag-tagged wtSTAT5B, STAT5B^N642H^ and endogenous STAT5A/STAT5B protein expression (*n* = 2). Actin served as the loading control.

Gain of function mutations of *STAT5B* have been described in hematopoietic malignancies. The recurrent hotspot mutation N642H has been identified in T cell leukemia and lymphomas and the STAT5B^N642H^ mutant was shown to induce T cell neoplasia in transgenic mice [24,25,26,27]. We therefore tested the ability of 17f to inhibit STAT5B^N642H^ expression and growth of hematopoietic cells transformed by this mutant. For this purpose, we used Ba/F3 cells expressing flag-tagged STAT5B^N642H^ or flag-tagged wild-type STAT5B (wtSTAT5B) as control [27]. We found that Ba/F3-STAT5B^N642H^ cells were more sensitive to 17f treatment than control Ba/F3-wtSTAT5B cells (Figure 8A). We then addressed whether STAT5^N642H^ expression was impacted by 17f and showed that 17f reduces expression of this mutant in Ba/F3 cells but does not affect wtSTAT5B or endogenous STAT5A expression after 24 h treatment (Figure 8B).

## 3. Discussion

The development of pharmacological inhibitors targeting the JAK/STAT pathway has been the subject of intense investigation during the last decade. Among the STAT family members, STAT5 proteins are now recognized as important therapeutic targets in hematologic malignancies and also in certain solid tumors [28]. Distinct pharmacological compounds that directly or indirectly affect STAT5 activity and leukemia cell growth have been used or developed during these last years. We recently synthesized and discovered a new compound (17f) that inhibits STAT5 phosphorylation and transcriptional activity in various CML and AML cells, without detectable effects on other signal transduction molecules, such as STAT3 and the protein kinases ERK1/2 and AKT [23]. We also demonstrated that 17f strongly reduces the growth of CML and AML cells with EC_50_ values below 10 µM close to EC_50_ values obtained with the STAT5 inhibitor pimozide (unpublished data) indicating that 17f as pimozide targets myeloid leukemia cells addicted to STAT5 signaling (see also Figure S1 for 17f and pimozide structures). In this study, we bring evidences that 17f also relieves the resistance of CML and AML cells to IM and Ara-C, respectively. Interestingly, we found that the concentrations of 17f required to restore the response to IM and Ara-C in resistant leukemic cells were much lower than EC_50_ values obtained for each resistant cell type. Indeed, inhibition of cell growth was already observed with 1 µM when combined with IM or Ara-C while EC_50_ values obtained for 17f compound alone were greater than 10 µM in these resistant cells. Depletion of IM or Ara-C in resistant cells might explain changes in the growth inhibitory effects of 17f. Indeed, we observed that the removal of IM strongly increases the phosphorylation of STAT5 in K562R cells. In these conditions, P-Y-STAT5 protein levels remain much higher in K562R cells after 17f treatment than in treated K562S cells, which are sensitive to lower concentrations of 17f. These data are in close agreement with a previously published study showing that high STAT5 levels mediate IM resistance in CML cells [8]. Although the removal of Ara-C results in a slight increase in STAT5 phosphorylation, the resistance of MV4-11R cells to this drug is not directly linked to overactivated STAT5. ERK1/2 and AKT kinases that also play a crucial role in cell survival, are involved in the resistance of MV4-11 cells to Ara-C [29]. It is then likely that Ara-C depletion may overexpress or overactivate these survival pathways in resistant MV4-11 cells. Whatever the resistance mechanism associated or not with STAT5 signaling, our data suggest that combination treatments with a STAT5 inhibitor might efficiently eliminate resistant CML and AML cells.

While 17f alone inhibited STAT5 phosphorylation in IM-depleted K562R cells, it decreased STAT5 expression in Ara-C-depleted MV4-11R cells. Importantly, combination treatments reduced expression of STAT5 in both resistant leukemic cells. The mechanisms involved in this downregulation remain unknown but are not associated with changes in *STAT5A* and *STAT5B* gene expression and specifically affect STAT5B protein. Importantly, we also demonstrated that 17f inhibits expression of STAT5B^N642H^ protein expression in transformed Ba/F3 cells. STAT5B^N642H^ is a driver mutation for T cell neoplasia and has been associated with aggressiveness, poor prognosis and an increased risk of relapse in T cell leukemia-lymphoma patients [24,25,26,27]. In addition to myeloid leukemia, 17f might be then employed to target lymphoproliferative disorders and lymphomas addicted to STAT5B^N642H^ signaling. 

It is likely that 17f inhibits STAT5B expression via the ubiquitin/proteasome-dependent degradation of this protein. Indeed, STAT5 proteins were previously shown to be ubiquitinated and several ubiquitination sites have been identified in STAT5A and STAT5B protein sequences [30,31]. Cbl, a well-known E3 ubiquitin ligase was found to interact with STAT5 and to induce its ubiquitination [30]. Moreover, cytokine-mediated STAT5 phosphorylation was enhanced in hematopoietic stem cells from *c-cbl* knockout mice [32]. 17f alone or associated with IM or Ara-C might then promote ubiquitination and proteasomal degradation of STAT5B protein in resistant leukemic cells as well as in STAT5B^N642H^-expressing cells. In a similar vein, pimozide was shown to target USP1, a ubiquitin specific protease involved in the deubiquitination of transcription factors such as ID-1. Pimozide-mediated inhibition of USP-1 promotes ID1 degradation and inhibition of leukemic cell growth [12]. It is therefore conceivable that 17f activity is connected to *a* proteasome regulatory network that controls STAT5B protein degradation. Alternatively, the combination of 17f and IM or Ara-C might also target chaperone molecules such as the heat shock proteins HSP90 or HSP70 proteins which were previously shown to regulate expression and/or stability of STAT5 [33,34]. The dual inhibition of BCR-ABL and HSP90 was shown to abrogate the growth of IM-resistant CML cells [35]. Furthermore, a key role of STAT5 has been demonstrated in the synergistic effects of FLT3 and HSP90 inhibitors in FLT3-ITD-expressing leukemic cells [36]. Importantly, HSP90 inhibitors not only target STAT5 but also overcome the resistance of AML cells to FLT3 inhibitors [37]. HSP70 was also found to induce STAT5 expression and drug resistance in AML and CML cells and inhibition of STAT5 activity was sufficient to resensitize resistant leukemic cells to chemotherapy [34,38].

If the downregulation of STAT5A and STAT5B expression can occur via ubiquitin/proteasome-dependent protein degradation, the selective effect of 17f on STAT5B still remains unclear. Nevertheless, using a bacterial two-hybrid screening approach, we previously identified the tumor suppressor hTid1 as a specific binding partner of STAT5B [39]. hTid1 belongs to the DnaJ chaperone protein family, which contains the J domain, a highly conserved domain that binds to Hsp70. The DnaJ-Hsp70 complexes are involved in protein folding and protein degradation and hTid1 was shown to promote the ubiquitination and degradation of various cellular proteins including transcription factors [40]. We demonstrated that overexpression of hTid1 specifically suppresses STAT5B protein expression and the transforming potential of a constitutively active STAT5B variant (STAT5B1*6) in hematopoietic cells. 17f might then target specific effectors of STAT5B protein stability/degradation, a hypothesis that has yet to be experimentally tested.

Besides these potential mechanisms, the capacity of 17f to restore the sensitivity of resistant CML or AML cells to IM or Ara-C suggests that inhibitors targeting STAT5 expression would also benefit AML or CML patients who have developed resistance to chemotherapy. Accordingly, PPARγ agonists were shown to inhibit *STAT5A* and *STAT5B* gene expression and to synergize with IM to eradicate resistant CML stem cells [10]. Our findings suggest that targeting STAT5B protein is a promising therapeutic strategy to eradicate leukemic cells that acquired resistance to chemotherapeutic agents. This is also supported by previous works showing that STAT5B but not STAT5A plays a key role in BCR-ABL-induced leukemogenesis and in the sensitivity of CML cells to TKI treatment [41,42]. Recent studies indicated that STAT5 proteins also exert important non canonical functions in normal and cancer cells. For instance, unphosphorylated STAT5 (uSTAT5: non phosphorylated on Y694/699 residues) were shown to be transcriptionally active in self-renewing hematopoietic stem cells and to promote leukemia/lymphoma cell survival [43,44]. Selective inhibitors that only block tyrosine phosphorylation and dimer formation might then be insufficient to fully abrogate STAT5 activity and resistance to chemotherapy. Herein, we showed that that inhibition of STAT5B expression elicited by 17f might unlock drug resistance in CML and AML cells. Using these promising data as a lead, we carried out a rational search for new derivatives of 17f with enhanced antileukemic activity. Modeling work was initiated to identify a pharmacophore that could help to optimize the development of 17f derivatives working in the nanomolar range. These new compounds could represent promising drugs to overcome chemotherapy resistance in leukemia or lymphomas. 

## 4. Materials and Methods 

### 4.1. Cell Cultures and Reagents

IM-sensitive (K562S) and IM-resistant (K562R) BCR-ABL^+^ cells and MV4-11 cells were obtained from American Type Culture Collection (ATCC) and Deutsche Sammlung von Mikroorganismens und Zellkulturen (DSMZ), respectively, and maintained according to the supplier’s recommendations. K562R and Ara-C-resistant MV4-11 (MV4-11R) cells were obtained after cultures of K562S and sensitive MV4-11 (MV4-11S) cells with increasing concentrations of IM and Ara-C (until 1 µM). Ba/F3-STAT5B^N642H^ and Ba/F3-wtSTAT5B cells were previously described in [27]. All cell lines were cultured in RPMI 1640, with 10% fetal bovine serum, 2 mM glutamine, 100 U/mL penicillin and 100 µg/mL streptomycin at 37 °C, 5% CO_2_. Resistant cells were cultured with 1 µM IM or Ara-C. IM was purchased from Selleckchem (Houston, TX, USA) and Ara-C from Sandoz France (Levallois-Perret, France). Ba/F3-wtSTAT5B were cultured with IL-3. The synthesis of the 17f compound was previously described in [23].

### 4.2. Cell Proliferation Assays

Cell viability and proliferation were studied using a MTT cell proliferation assay (Sigma-Aldrich, St Louis, MO, USA). Briefly, 2 × 10^4^ leukemic cells were cultured in 100 µL of RPMI medium in 96-well plates and treated with drugs for 48 h. Cells were then incubated with 10 µL of MTT working solution (5 g/L of methylthiazolyldiphenyl-tetrazolium bromide) for 4 h. Cells were lysed overnight at 37 °C with 100 μL of SDS 10%, HCl 0.003%. Optical density (OD) at 570 nm was then measured using a spectrophotometer CLARIOstar^®^ (BMG Labtech, Offenburg, Germany). Living cells were also enumerated using the trypan blue dye exclusion method.

### 4.3. Apoptosis and Cell Cycle Analysis

Cells were washed with PBS, then stained (10^6^ cells) in buffer containing FITC-annexin V and 7-amino-actinomycin D (7-AAD) (Beckmann Coulter, Fullerton, CA, USA) for 15 min at 4 °C and analyzed by flow cytometry (Becton Dickinson Accuri™ C6 flow cytometer). For cell cycle analysis [45], cells were first incubated with fixing solution (PFA 2%, Hepes 1%, saponin 0.03%) for 15 min and then in PFS permeabilization solution (PBS 1×, SVF 10%, saponin 0.03%, Hepes 1%). Cells were next stained for 30 min at room temperature with anti-Ki67-Alexa Fluor 488 monoclonal antibody or the corresponding isotype as control (Becton-Dickinson, Franklin Lakes, NJ, USA) before analysis by flow cytometry (Becton Dickinson Accuri™ C6 flow cytometer). The FlowJo^®^ software (V10.1, BD Biosciences, Franklin Lakes, NJ, USA) was used to analyze data. 

### 4.4. Plasmids, Transfection and Luciferase Reporter Assays

The 6×(STAT5)-TK-luc containing six tandem copies of the STAT5 binding site linked to the minimal TK-luciferase reporter gene and control TK-luc plasmids have been described elsewhere [46]. For transient transfection assays, cells were electroporated (270 V, 950 μF) with the different constructs (50 μg). Transfected cells were expanded for 24 h in medium and then treated for 48 h. Cell extracts were then prepared in luciferase buffer according to the manufacturer’s protocol (One Glo luciferase assay kit, Promega, Madison, WI, USA). Luciferase activities were measured in a luminometer CLARIOstar^®^ (BMG Labtech, Offenburg, Germany).

### 4.5. Western Blot

Cells were suspended in Laemmli’s 2× buffer (Bio-Rad, Hercules, CA, USA), separated on SDS/PAGE and blotted onto nitrocellulose membrane. Blots were incubated with the following antibodies (Abs): P-Y^694/699^-STAT5, Actin (Cell Signaling Technology, Danvers, MA, USA), STAT5 (BD Transduction Laboratories, Franklin Lakes, NJ, USA), STAT5A and STAT5B (Zymed/ThermoFisher Scientific, Waltham, MA, USA). Membranes were developed with the ECL chemiluminescence detection system (GE Healthcare, Little Chalfont Buckinghamshire, UK) using specific peroxidase (HRP) conjugated to rabbit or mouse IgG antibodies (Cell Signaling Technology). 

### 4.6. P-Y^694/699^-STAT5 Flow Cytometry Analysis

Cells were washed in PBS and incubated with a fixing solution PFA 4% for 15 min at room temperature. The first permeabilization solution PBS/Triton X-100 0.2% was then added and incubated for 30 min at 37 °C. After being washed with PBS/BSA 0.5%, cells were suspended with the second permeabilization solution PBS/MeOH 50% and incubated for 10 min on ice. Cells were then stained with anti P-Y^694/699^-STAT5 antibodies or the corresponding isotype as control (BD Biosciences, NJ, USA) for 30 min at room temperature before analysis by flow cytometry (FACS Canto II, BD Biosciences).

### 4.7. qRT-PCR Analysis

RNA samples were reverse-transcribed using the SuperScript^®^VILO cDNA synthesis kit (Invitrogen, Carlsbad, CA, USA) as recommended by the supplier. The resulting cDNAs were used for quantitative real-time PCR (qRT-PCR). PCR primers (*PIM1*: *for 5′-*TTTCGAGCATGACGAAGAGA-3′, *rev* 5′-GGGCCAAGCACCATCTAAT-3′; *CISH*: 5′- AGCCAAGACCTTCTCCTACCTT-3′, *rev* 5′-TGGCATCTTCTGCAGGTGT-3′; *STAT5A*: *for* 5′-TCCCTATAACATGTACCCACA-3′, *rev* 5′-ATGGTCTCATCCAGGTCGAA-3′; *STAT5B*: *for* 5′-TGAAGGCCACCATCATCAG-3′, *rev* 5′-TGTTCAAGATCTCGCCACTG-3′) were designed with the ProbeFinder software (Roche Applied Sciences, Basel, Switzerland) and used to amplify the RT-generated cDNAs. qRT-PCR analyses were performed on the Light Cycler 480 thermocycler II (Roche). *GAPDH* (glyceraldehyde-3-phosphate dehydrogenase), *ACTB* (actin beta) and *RPL13A* were used as reference genes for normalization of qRT-PCR experiments. Each reaction condition was performed in triplicate. Relative gene expression was analyzed using the 2^−ΔΔCt^ method [47]. 

## 5. Conclusions

In summary, this work shows for the first time that inhibition of STAT5B expression might be a promising targeting strategy to bypass the resistance of CML and AML cells to TKI or conventional chemotherapeutic agents. Investigations to elucidate the mechanisms involved in STAT5B downregulation induced by these combination therapies might help to design new inhibitors that specifically target cancer cells addicted to oncogenic STAT5B signaling.

## Supplementary Materials

The following supplementary figures are available online at https://www.mdpi.com/2072-6694/11/12/2043/s1, Figure S1: Pimozide and 17f structures, Figure S2: Original western blot, Figure S3: Quantification of Western Blot data. Figure S4: Flow cytometry analysis of P-Y-STAT5 in K562S and K562R cells. Figure S5: Effects of 17f on P-Y-STAT5/STAT5 expression in IM-depleted K562R and Ara-C-depleted MV4-11R cells.
