# Supplementary material for: A Novel Inhibitor of STAT5 Signaling Overcomes Chemotherapy Resistance in Myeloid Leukemia Cells
Source: Cancers (Basel). 2019 Dec 17;11(12):2043. doi: 10.3390/cancers11122043 (PMC6966442; doi:10.3390/cancers11122043)
Supplement: Supplementary file 1 [file cancers-11-02043-s001.pdf]

Supplementary Materials:

A Novel Inhibitor of STAT5 Signaling Overcomes Chemotherapy Resistance in Myeloid Leukemia Cells

Marie Brachet-Botineau, Margaux Deynoux, Nicolas Vallet, Marion Polomski, Ludovic Juen, Olivier Hérault, Frédéric Mazurier, Marie-Claude Viaud-Massuard, Gildas Prié and Fabrice Gouilleux

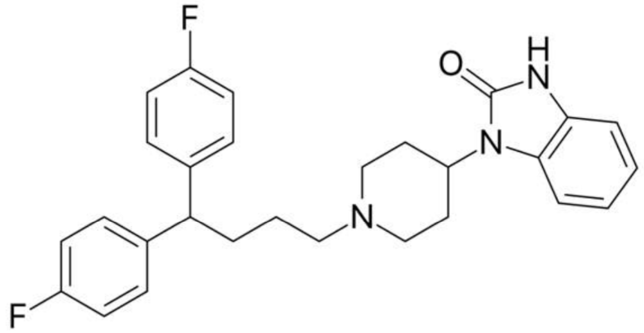

Pimozide
Mw = 461.56 g/mol

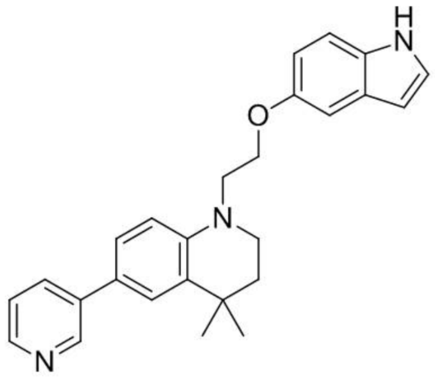

17f
Mw = 397.45 g/mol

Figure S1. Pimozide and 17f structures.

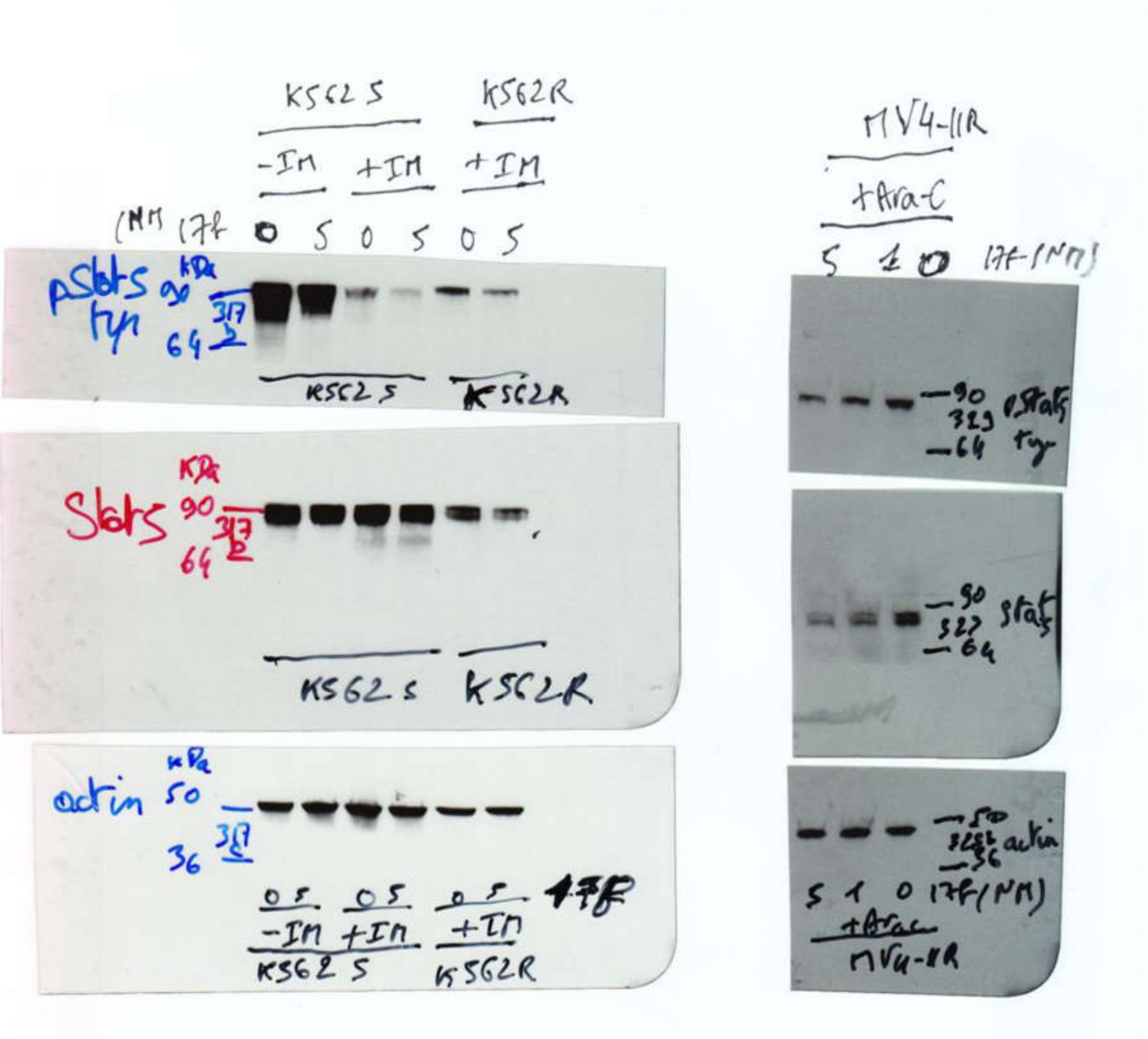

Figure S2. Original western blot data. Membranes were cut for probing with anti-P-Y-STAT5, anti-actin antibodies. Molecular weight markers (kDa) are indicated.

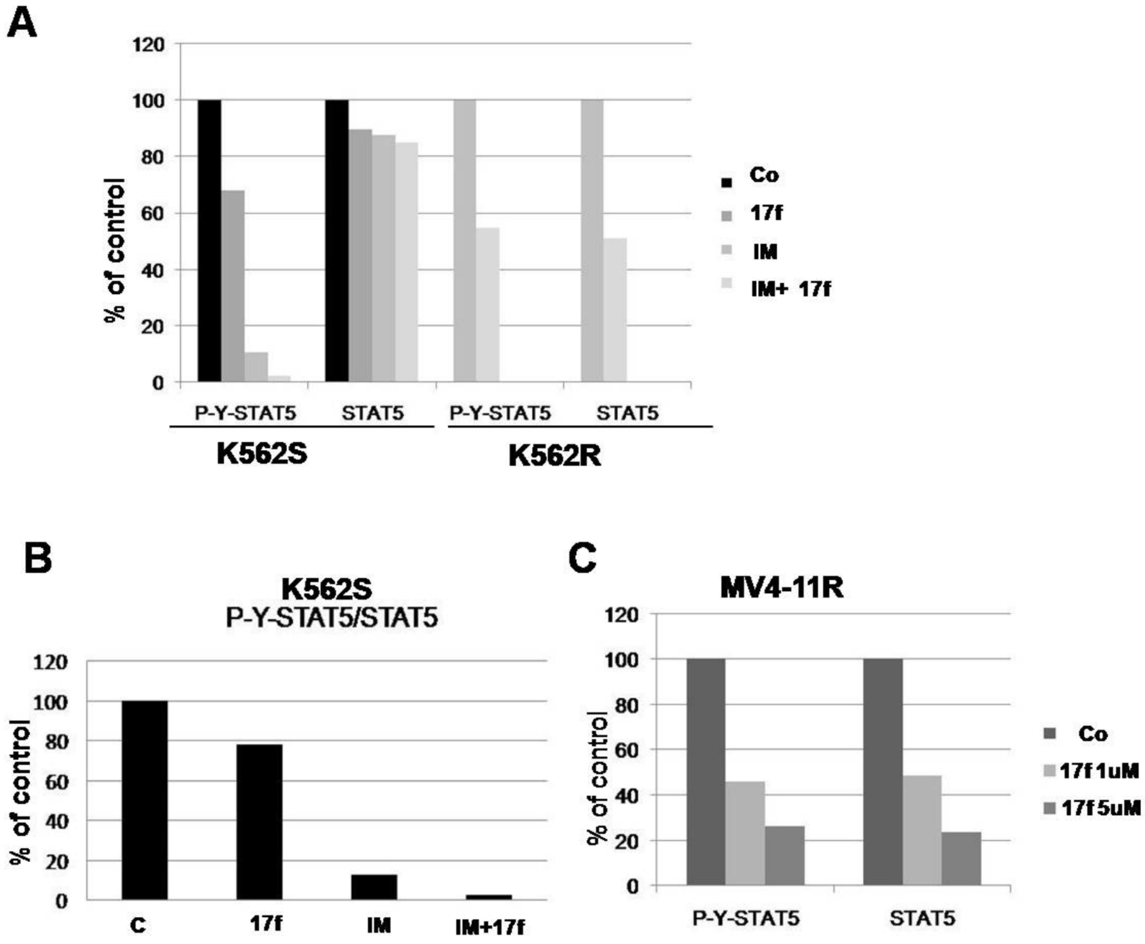

Figure S3. Quantification of Western Blot data (*n* = 2). **A**) Levels of P-Y-STAT5 (P-Y-STAT5/actin) and STAT5 (STAT5/actin) in K562S and K562R cells were evaluated by band intensity quantification (ImageJ software). For K562R cells, Controls are cells cultured with IM. **B**) P-Y-STAT5/STAT5 ratios were also determined in K562S cells following treatment with 17f, IM or a combination of both drugs. **C**) Levels of P-Y-STAT5 and STAT5 in MV4-11R cells were calculated as in (A).

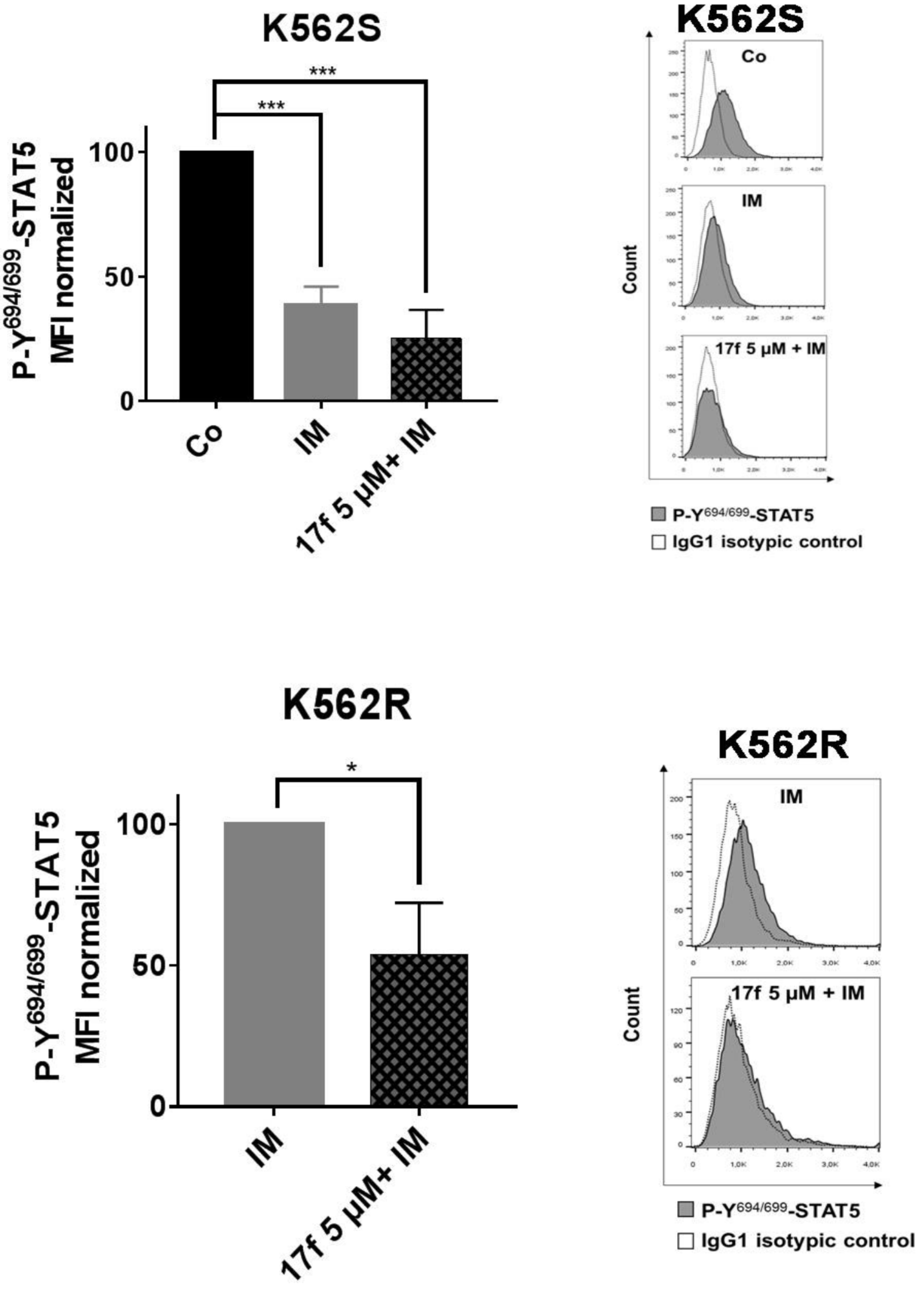

Figure S4. Flow cytometry analysis of P-Y-STAT5 in K562S and K562R cells. Cells were treated (IM) or not (Co) with or without 17f for 24 h. MFI values in the histogram represent the mean fluorescence intensity (MFI). Representative experiments are shown (right panel) ($n = 3$ in triplicates, data are mean ±SD, * $p < 0.05$, *** $p < 0.001$; one-sample *t*-test).

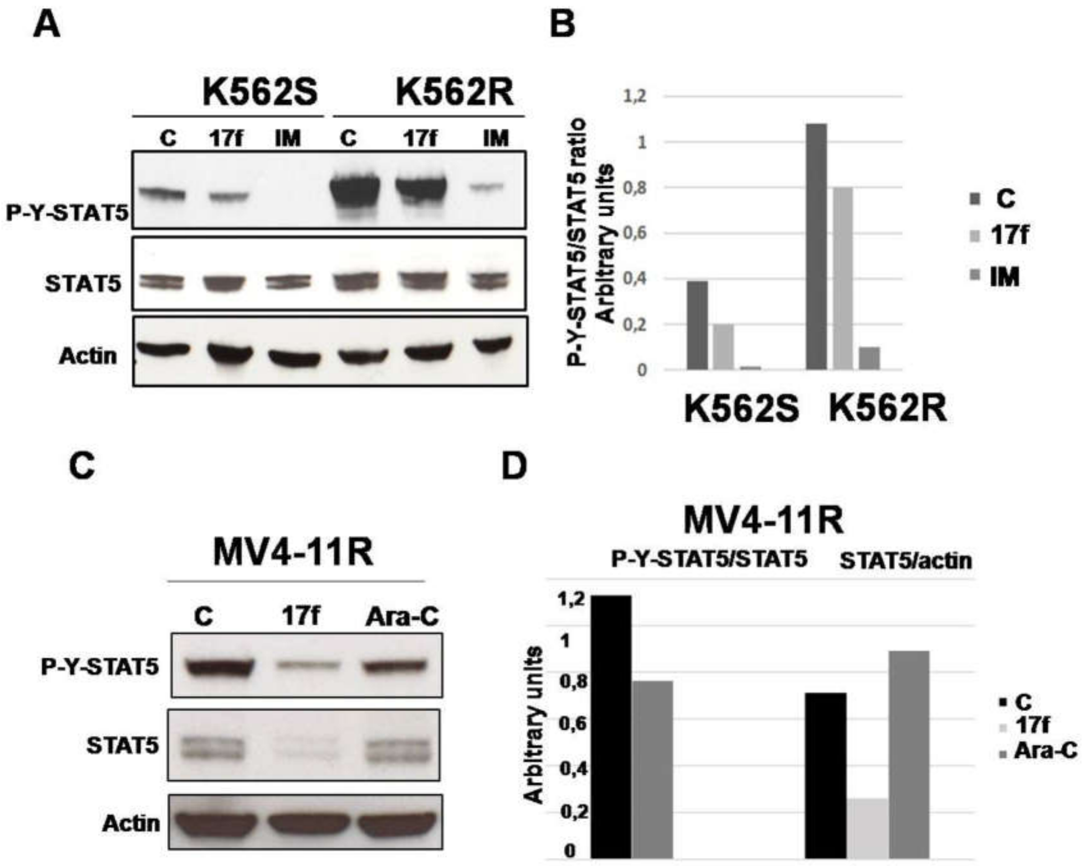

Figure S5. Effects of 17f on P-Y-STAT5/STAT5 expression in IM-depleted K562R and Ara-C-depleted MV4-11R cells. **A**) Protein extracts form K562S and IM-depleted K562R cells treated with 17f (5 μM), IM (1 μM) or DMSO as control or 24 h were analyzed by western blot to detect P-$Y^{694/699}$-STAT5/STAT5 and STAT5 protein expression (*n* = 2). Actin served as loading control. **B**) P-Y-STAT5/STAT5 ratios were also calculated in K562S and IM-Depleted K562R cells after treatment or not with 17f or IM **C**) Ara-C-deleted MV4-11Rcell lysates treated or not (C) with 17f (5 μM) or Ara-C (1 μM) for 24 h were analyzed by western blot to detect P-$Y^{694/699}$-STAT5 and STAT5 protein expression (*n* = 2). Actin served as loading control. **D**) Quantification of western blot data form C.

© 2019 by the authors. Licensee MDPI, Basel, Switzerland. This article is an open access article distributed under the terms and conditions of the Creative Commons Attribution (CC BY) license (http://creativecommons.org/licenses/by/4.0/).
